# Barriers and facilitators to implementation of the Accountable Health Communities (AHC) Model: Findings from a between-site qualitative assessment of implementation strategies

**DOI:** 10.3389/frhs.2022.926657

**Published:** 2022-11-02

**Authors:** Linda Highfield, Gayla M. Ferguson, Jennifer Holcomb

**Affiliations:** ^1^Department of Management, Policy and Community Health, The University of Texas Health Science Center at Houston (UTHealth) School of Public Health, Houston, TX, United States; ^2^Department of Epidemiology, Human Genetics and Environmental Sciences, The University of Texas Health Science Center at Houston (UTHealth) School of Public Health, Houston, TX, United States; ^3^Department of Internal Medicine, The University of Texas Health Science Center at Houston (UTHealth) John P. and Katherine G. McGovern Medical School, Houston, TX, United States; ^4^Sinai Urban Health Institute, Sinai Chicago, Chicago, IL, United States

**Keywords:** Consolidated Framework for Implementation Research, Accountable Health Communities Model, implementation barriers and facilitators, cross-site analysis, essential elements

## Abstract

**Introduction:**

A multitude of HRSN interventions are undergoing testing in the U.S., with the CMS Accountable Health Communities (AHC) Model as the largest. HRSN interventions typically include screening for social needs, referral to community resources, and patient navigation to ensure needs are met. There is currently a paucity of evidence on implementation of HRSN interventions. The Consolidated Framework for Implementation Research (CFIR) is a determinant framework widely used to plan and assess implementation. To the authors knowledge, there are no published studies assessing CFIR constructs for HRSN intervention implementation in the U.S. In the Assessment step of the Strengthening Peer AHC Navigation (SPAN) model, a between-site qualitative assessment methodology was used to examine implementation within and between AHC bridge organizations (BOs) within six ERIC implementation strategies identified by the authors based on AHC Model requirements.

**Objective:**

Our aim was to identify and present between-site barriers and facilitators to AHC Model implementation strategies.

**Design:**

A multi-site qualitative analysis methodology was used. CFIR determinants were linked to six Expert Recommendations for Implementing Change (ERIC) strategies: staff training, identify and prepare champions, facilitation, community resource engagement (alignment through advisory boards and working groups), data systems, and quality monitoring and assurance. Interviews were analyzed using thematic content analysis in NVivo 12 (QSR International).

**Setting:**

Five health-related bridge organizations participating in the AHC Model.

**Results:**

Fifty-eight interviews were completed with 34 staff and 24 patients or patient proxies. Facilitators were identified across five of the six ERIC strategies. Barriers were identified across all six. While organizations found the AHC Model compatible and facilitators to implementation included previous experience, meeting patient needs and resources, and leadership engagement and support, a number of barriers presented challenges to implementation. Issues with adequate staff training, staff skills to resolve HRSN, including patient communication and boundary spanning, setting staff goals, beneficiary caseloads and measurement of progress, data infrastructure (including EHR), available resources to implement and differences in perceptions between clinical delivery site (CDS), and CSP of how to measure and resolve HRSN.

**Conclusions and relevance:**

The conduct of a pre-implementation readiness assessment benefited from identifying CFIR determinants linked to various ERIC implementation strategies.

## Background

Health-related social needs (HRSN), particularly lack of stable housing, consistent access to food, transportation, paying utility bills, and a safe environment, impact population health, healthcare utilization, and costs (1). Health systems across the U.S. recognize the impacts of HRSNs and rapid testing of interventions has followed (2). HRSN interventions implemented to date typically include screening, referral to community resources, and patient navigation to facilitate connection with resources (2). While early findings are emerging (3–6), there is a paucity of evidence using the implementation science methods available, leaving practitioners with little guidance on how to implement HRSN interventions. Studies to date have assessed patient and provider perceptions of acceptability, but none have considered broader domains and constructs including organizational, inter-organizational, community, and policy factors known to influence implementation. Recently published perspectives have noted this gap and highlighted documented barriers to implementation of cross-sector approaches like the AHC Model from previous studies, including differences across community service providers, insurance providers, and healthcare sectors in their approach to staffing, priorities, language and culture, financing and power (7). Damshroder et al. developed the Consolidated Framework for Implementation Research (CFIR) from 19 theories of dissemination, implementation, and organizational change (8). CFIR is a comprehensive determinant framework with 39 constructs organized into five domains that are relevant for assessment of implementation of cross-sector interventions: Intervention Characteristics, Characteristics of Individuals, Inner Setting, Outer Setting, and Process of Implementation (8). CFIR has been used to elucidate barriers and facilitators to implementation of interventions in a variety of studies and settings (9–12). However, to the authors' knowledge, there are no published studies available assessing CFIR domains and constructs for HRSN interventions in the U.S., nor are there any studies which assessed CFIR domains and constructs within the Expert Recommendations for Implementing Change (ERIC) implementation strategies.

The Centers for Medicare and Medicaid Services (CMS) conducted the largest-scale test of HRSN intervention in the U.S., through the Accountable Health Communities (AHC) Model which consisted of two Model Tracks, Assistance and Alignment, making the AHC Model an ideal setting to assess implementation (see https://innovation.cms.gov/innovation-models/ahcm) (13). In the Assistance Track of the model, social needs screening, referral to community resources and patient navigation for five core HRSNs (housing instability, food insecurity, transportation problems, utility help needs, and interpersonal safety) for eligible Medicare, Medicaid, and dually covered beneficiaries was conducted with a goal of resolved HRSNs within 1 year. The Alignment Track combined screening, referral, and patient navigation with engagement with key community stakeholders through advisory boards and continuous quality improvement, with the goal of ensuring that community services were available and responsive to address HRSNs (e.g., there were enough food pantries in the community to deliver needed food resources). In both the Assistance and Alignment Track of the model, Bridge Organizations (Bos) applied implementation strategies which were linked to Expert Recommendations for Implementing Change (ERIC) strategies: staff training, identify and prepare champions, facilitation, data systems, and quality monitoring and assurance. Alignment Track BOs also applied an additional strategy: community resource engagement (alignment through advisory boards and working groups) (13). BOs were expected to tailor and apply these implementation strategies in clinical delivery sites (screening, referral, and navigation) and community service providers (CSPs)/communities based on their model track. The implementation strategies BOs applied fit into ERIC strategies which were identified by the authors based on knowledge of the AHC Model and review of BO's standard operating procedures (SOPs) agreed upon by participating sites with CMS against the ERIC compilation (14). Based on the standard operating procedures required by CMS, all Bridge Organizations were expected to tailor and apply [the six] strategies for their specific communities. To allow for tailoring, CMS did not provide specific requirements on how to apply these. The mechanism, frequency, and dose in which these strategies were applied was at the Bridge Organization's discretion. For example, CMS required training of staff as part of the model. However, CMS did not specify what training should consist of (didactic or other content), how frequently it should be delivered, by whom, modality of delivery, nor dosage or follow-up measurement as each Bridge Organization tailored these for their communities.

Literature shows, however, that most organizations lack the skills needed to successfully apply these ERIC implementation strategies without expert technical assistance (TA) (15, 16). Mirroring this, technical assistance (TA) was identified as a priority need by BOs, indicating the AHC Model and its eventual evaluation may be affected by the presence of uneven capacity and readiness to execute and sustain a successful implementation plan (15).

The Strengthening Peer AHC Navigation (SPAN) protocol was developed as a multi-level quality improvement (QI) intervention to improve AHC Model implementation and provide technical assistance for five BOs in the AHC Model. A full description of the SPAN protocol can be found elsewhere (17). The first of four tasks in SPAN was a within-site assessment of each Bridge Organization's current implementation, TA needs, and readiness in the AHC Model (17). To identify commonalities in barriers and facilitators to implementation strategies across the five sites, a between-site qualitative assessment methodology using CFIR domains and constructs was conducted within each ERIC strategy. Our focus in this study is on the real-world application of these ERIC strategies by each of the Bridge Organizations and identifying the cross-cutting barriers and facilitators they encountered in applying the strategies when provided general guidance for model implementation.

## Methods

### Within-site qualitative analysis

Semi-structured patient and staff interviews were conducted from May 2020 to May 2021 (17). Domains assessed in the staff and patient interview guides have been reported elsewhere (17). Briefly, CFIR constructs were used to assess barriers and facilitators to implementation. The interview guide was developed using sample interview questions available on http://cfirguide.org/ which were tailored for AHC Model activities [see Holcomb et al. (17) for the published interview guides] (17). Purposive sampling was used to identify staff and patients for interviews to identify those with experiences in the AHC Model. We expected to interview at least three staff members and five patients from each bridge organization (17). Staff interviews were 60 min in length and conducted over WebEx video conference. Patient interviews were 30 min in the length and conducted over the phone. A caregiver or parent answered as a proxy for patients under 18 years old. Interviews were audio recorded by the lead interviewer, transcribed verbatim using a professional service, and then transcripts were checked for accuracy by the lead coder. The coding team included the lead coder, two secondary coders, and an outside reviewer for the multi-site qualitative analysis (18, 19). The team was comprised of female qualitative and mixed methods researchers in an academic setting. The lead coder and interviewer had no relationship with the participants. Two coding team members knew the interviewees through model interactions. Interviewees were provided a description of the study and interest by the lead interviewer prior to consent. Interviews were analyzed using thematic content analysis using NVivo 12 (QSR International).

The coding team developed a codebook for staff interviews and a modified codebook for patient interviews based on the CFIR qualitative coding guide. Operational codes for CFIR determinants were the first level of coding. The CFIR codes were analytical and required each coder to interpret the data and then apply the CFIR code that reflected a barrier or facilitator (19–21). To ensure comprehensive coding and no loss of data, open and adaptive codes were also used. Following first-level coding, a second level analysis was done to align identified CFIR codes with the six components of the AHC Model. The three coders used the codebooks and coded each BO independently. The coders met weekly to discuss individual coding, emerging patterns, and common themes. Themes from staff interviews were organized by barriers and facilitators in the intervention across the six AHC Model components identified by the research team and CFIR constructs for each BO. Themes from the patient interviews were then compared to staff interviews and any differences between staff and patient themes were discussed by the coding team until consensus was reached on BO specific themes. Analytic matrices or tables (see Supplementary Table) of barriers and facilitators were created for each BO (22). Each analytic matrix was reviewed with the participating BOs and clinical delivery site (CDS) staff for accuracy. This ensured that the CFIR constructs and barrier/facilitator themes that the coders identified aligned with the BO and CDS areas of focus for QI in the site's current implementation approach and were feasible for implementation of a QI plan. A full description of this step in the SPAN intervention and the complete SPAN protocol can be found elsewhere (17).

### Between-site qualitative analysis

**Figure 1:**
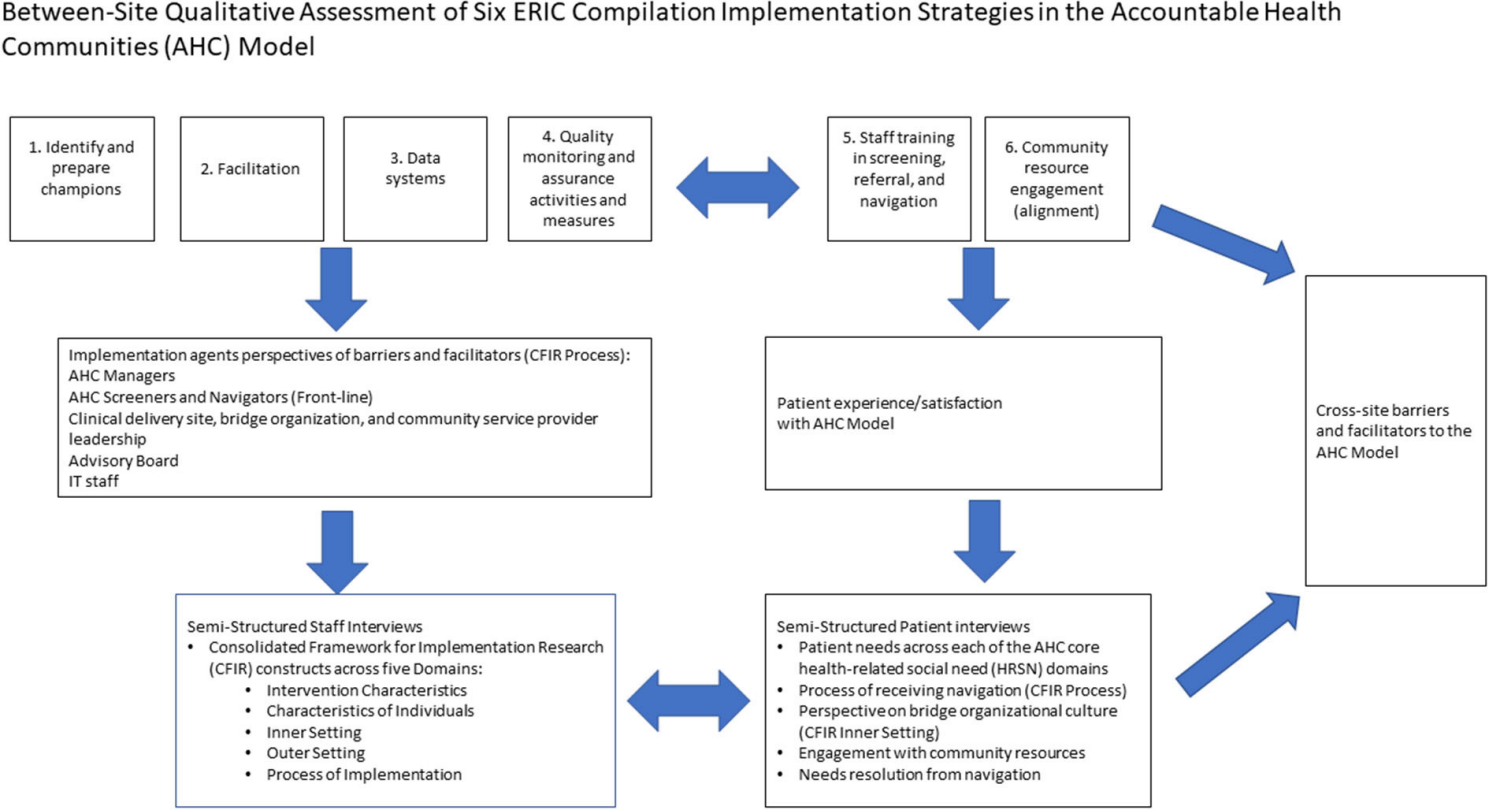
Conceptual framework for assessment of implementation strategy barriers and facilitators.

In this step of the analysis, the team used a similar analytic matrix from the Within-Site Qualitative Analysis, which allowed for the coding of two CFIR domains and constructs per model component. First, the team individually reviewed the BO specific analysis and completed the matrix independently. The coding team met and discussed each person's perspective of the BO themes. This was to ensure that context was preserved for between- site coding. The team reviewed each coder's individually developed between-site matrix. In cases where chosen CFIR domains and constructs did not align across the team, discussion was used to ensure understanding of the selection for all coders. Following discussion, each coder voted independently to select the final two CFIR domains and constructs per model component (12). This process aligned with the flexibility of the CFIR as a “menu” to be tailored to a specific context and allowed coders to narrow down the CFIR domains to those most reflective of the data (12). The team then collectively drafted two over-arching barrier and facilitator themes for each model component. Draft themes were formatted into the matrix. The interview transcript data from the full coded transcripts of all sites were reviewed to identify patient and staff quotations to illustrate between-site barriers and facilitators. This ensured all interview data were used to generate between-site themes. This process ensured that the overarching themes accurately represented the data and preserved context. The between-site qualitative assessment of the six ERIC implementation strategies in the AHC Model is shown in Figure 1.

## Results

Fifty-eight interviews were completed with the five participating BOs. The five BOs represented a range of health-related organizational types (17). Thirty-four interviews were conducted with BO, CDS, and CSP, staff including leadership, managers, front-line implementers, and advisory board members. Interviews were mostly conducted with AHC program manager and navigation supervisors (*n* = 13) with 10 each conducted with leadership (Vice President, Director) and front-line implementers (CHWs, navigators) and one with an advisory board member. Twenty-four patients or patient proxies were interviewed, with 19 reporting demographic information. The average age of patients was 37 years old (range 2–75 years). Patients mostly identified as female (*n* = 15; 78.9%), lived in a household with <$20,000 in annual income (*n* = 12; 63.2%) and had less than a high school degree or GED (*n* = 15; 78.9%). Three patients were Non-Hispanic Black or African American (15.8%), four Non-Hispanic Asian (21.1%), one identified as Human, four were Non-Hispanic White (21.1%), and seven were Hispanic (36.8%). All patients had been screened for HRSNs, referred to community resources, completed a personal interview about HRSNs with navigation staff, and had been contacted at least once in a follow-up from a navigator. Cross-cutting between-site facilitator and barrier CFIR determinant themes are summarized below by the six model components by CFIR domain (e.g., Characteristics of Individuals).

### Staff training

#### Characteristics of individuals (individual level)

Staff indicated *Self-efficacy* was both a facilitator and barrier in implementation. Staff, particularly Community Health Workers (CHWs), were confident working with patients on HRSNs. “*…our goal is that anyone who enters our organization through …any modes of our programs…we help them navigate their social determinants of health that…they've identified, and help them to live a better and healthier life” (Staff quote)*. Staff, however, lacked the *self-efficacy* to shift iteratively from analyzing the patient's situation, planning and providing resources to meet their needs, applying knowledge and skills tailored to each beneficiary during navigation, and evaluating the impact on HRSN resolution. The resolution of housing and transportation HRSNs was particularly difficult due to this lack of boundary spanning skills. “*…I did ask…[the navigator] if [they] knew of anything because they always made it sound like they knew more resources that were already available, and I explained that I…was very well aware of the resources that were available. But I was looking for something outside of [211] …the resources that they were giving me were all the basic resources that I already knew of” (Patient quote)*.

#### Inner setting (organizational level)

*Access to knowledge and information* as a barrier was reflected by a lack of a ready BO workforce with sufficient training and boundary spanning capabilities to address and resolve HRSNs and to apply evidence-based patient engagement and communication strategies. “*I think what I see as a gap in skill at this point is more related to motivational interviewing…. What I see is that our team understands the spirit of it—they know the basics—but have… a difficult time applying some of the more advanced techniques…so they can do reflections. They can listen emphatically…It's more so getting to that ambivalence, listening for the change talk, and really working with people at that higher level. I see that as a skillset, which is something I [would] like to see us improve because it may be able to lengthen our engagement. I eventually see more resolution of needs. Because at this point, I feel like there are some [patients] who really engage deeply over a long period of time…” (Staff quote)*.

### Identify and prepare champions

#### Inner setting

Through *Leadership Engagement*, managers felt they could share program updates and in turn leadership listened and provided a supportive environment overall to facilitate implementation. “…*working with the load that we have, it can become overwhelming. But when you have…[an] organization that cares about your work— about us and the work that we do, it definitely makes it a lot easier to be able to assist our [patients]” (Staff quote)*. The *leadership engagement* sub-construct was also a barrier. BOs encountered difficulty gaining buy-in, support, and engagement from clinical site leadership, especially at the executive level, partially due to turnover in AHC liaisons at the CDS. BO staff also perceived that hierarchical organizational structures prevented effective communication from leadership to the front-line staff. “*…a lot of agencies we work with…have a very like top-down management style…And then a lot of times, especially in a hospital system, I think that messaging gets a little bit lost along the way, especially when the person who's asked to do the project is maybe their equivalent of a minimum wage worker or maybe someone who does get paid the least amount within that hospital system…I think some of the connection between the higher management levels and the folks doing the work has been lost sometimes” (Staff quote)*.

### Facilitation

#### Inner setting

Another facilitator for implementation was the AHC Model being *Compatible* with existing BO climate, workflow, and staff roles. Organizations reporting previous experience with social needs programming found the AHC Model to align with their experience, thereby making it easier to facilitate implementation in the clinical delivery setting. “*…we already had a similar screening process in place already… I do think that it was a great addition to what we already do because it was backup to make sure that these patients that were high-risk coming into those ER visits were actually contacted [for navigation] …. make sure that they were reached out [to]...” (Staff quote)*.

As front-line implementers worked with patients to resolve needs, model, BO, and individual staff requirements did not always align with the demand and urgency of patient needs creating difficulty in implementation facilitation. Screeners and navigators reported feeling a disconnect between the goals communicated by managers and leadership with their experiences as reflected in *Networks and Communication*. “*…these are individuals that are struggling…whether it's food or a place to live... They can't wait two weeks and follow up a month later. We need to be on it…a lot faster…which to me, that would have been a core measure that should have been a milestone…, our resolution rate and timeliness of resolution…. We can't take up to a year to resolve their problem. I understand housing waitlists, but food insecurity, there are opportunities to provide food within probably the same day, at the very least within the next day. If they're in a desperate situation, we're not going to wait two weeks to help them…That would be a more meaningful metric than [X number of] screenings” (Staff quote)*. This urgency to meet needs were also represented by patient stories. “*…I was in a very difficult spot, but they didn't call me until like four months later…I said, “You were supposed to call me the next day.”…By the time that [they] called me, I didn't even need the resources anymore” (Patient quote)*.

### Data systems

#### Inner setting

An *Available Resource* facilitator was existing data system infrastructure. BOs and CDS utilizing existing case management systems were comfortable using software to navigate patients and this reduced time needed for training, duplicative data entry, and data errors, and improved reporting. “*…we were the pilot organization for [a] case management system…we already had a pretty close relationship with them… it was just a natural progression that we would be involved with this as well because it did a lot of the same things that we were already testing” (Staff quote)*.

#### Inner setting

A lack of *available resources* in funding for data systems and IT infrastructure, integration of electronic health record (EHR) and AHC data, and lack of experience in database management further complicated implementation. This led to duplication of data entry, opportunities for data entry errors, and a lack of timely data collection and reporting. “*…we have staff trying to basically do duplicate work, and part of that was because [the AHC data system] does not feed directly into [the EHR] it's not like we can put the data in there, which quite honestly would help with a better compliance if it was directly tied to our healthcare system in the [EHR] usage. I think we'd actually have a higher compliance because it makes it part of the screening questions…during this person's intake” (Staff quote)*.

### Development of quality monitoring and assurance

#### Inner setting

*Networks and Communication were a facilitator and f* ostered mid-level staff integration of the AHC Model in the clinical setting. Managers monitored *goals* and provided *feedback* to staff in a timely and actionable manner which supported staff integration of AHC processes into their daily workflows. Managers in this climate were innovative and tested strategies including multiple screeners (i.e., medical assistants, CHWs, front desk staff, navigators) and screening modes (i.e., phone, paper, tablet) which allowed managers to create tailored navigation milestones, while maintaining the required boundaries of the AHC Model. “*And so we do now have a weekly target for each navigator, saying, “Based on your current caseload, this is the number we expect you to hit. This is your goal to work towards.” And I think that's been really great for them, so they feel like it's very tailored to them rather than this overarching goal they're like, there's no way I'll hit that because of the patients I have that…” (Staff quote)*.

#### Inner setting

Challenges with data systems created barriers in *Goals and Feedback and Tension for Change*. Managers found it difficult to measure the impact, return on investment, and value of the AHC Model with limited real-time data. “*…What is our successful resolution rate? From the AHC evaluation… our rate is higher. Are we looking at this right? Is our resolution rate fine?*

*Should we try to improve it? What does this data mean? Are we doing well? I don't know what to compare it to” (Staff quote)*.

### Community resource engagement (alignment of patient needs and available, accessible community resources through advisory boards and working groups)

#### Characteristics of individuals (individual level)

*Other personal attributes* of individuals represented a barrier to community alignment. The ability and capacity of mid-level management (Program/project managers) to effectively lead and facilitate their advisory boards were limited. Challenges in recruitment and engagement of advisory boards was also a barrier. “*… there's still more that I could learn from in terms of successful engagement, especially for those who don't want to engage either due to time or understanding the project or understanding of equity…. I think always there's good things to learn from engagement…I don't know if some of that goes into coaching or just—I guess the equivalent of how do teachers get trained to work with difficult students would be like—that's what I want to know” (Staff quote)*.

#### Inner setting (organizational level)

*Available resources* were overall limited for community resource alignment. Staff time was limited and focused toward reporting on required model milestones. This was coupled with the lack of institutional resources to bolster capacity for alignment. Adequate time, training, and budget resources were not planned or allocated in the implementation design step by alignment BOs for formative advisory board activities. “*It takes a long time to not only get the community to recognize the value of collective impact, but the hospital and the healthcare workers to actually understand the value of true integration and working side by side” (Staff quote)*.

#### Outer setting

In addition, community-level resource alignment including sharing of funds was limited. This limitation was perceived by both staff and patients as a barrier to implementation and impacted *patient needs and resources*. “*…one of the other challenges is getting buy-in from the clinical delivery sites and CBOs and trying to make them understand why it would be of benefit to them to participate and to remain engaged… because there's not really always a financial incentive or something and there is extra work to be done by individuals and by sometimes volunteers and things. So, trying to impress the value of the program initially without having any kind of evidence or proof as to why it was a benefit, I think that's a challenge” (Staff quote)*. This alignment was important for introducing and navigating patients to community resources. “*...people don't know where to look; people don't know what to do...even though they gave me the resources..., I was repeatedly turned down because there just wasn't enough [funding] or I didn't meet the requirements... We really wish our system was set up better for people like [me]” (Patient quote)*.

## Discussion

This study sought to identify cross-site facilitators and barriers to implementation of the AHC Model through assessment of CFIR determinants across six ERIC implementation strategies from implementers (e.g., front-line staff, leaders). To our knowledge, no studies of the application of ERIC implementation strategies related to application of HRSN interventions have been published. Providing detailed data on determinants and barriers and facilitators to the application of ERIC implementation strategies is essential to further the field of HRSN intervention in the U.S (23). Previous studies have identified a broad set of pre-condition and enabling condition constructs that improve implementation for intersectoral interventions but have not linked these with specific determinant measures. These conditions include readiness, trust, sufficient community assets and capacity, history of program delivery, health system involvement, and the ability of a backbone organization to facilitate these conditions in their community (24).

In staff training, we found *self-efficacy* and *available resources* under Characteristics of Individuals were both barriers and facilitators. The first AHC Model evaluation report was developed to provide the public with early data on Medicare and Medicaid beneficiaries eligible for the AHC Model through December 2019. The report included beneficiary sociodemographic characteristics, HRSNs identified, participation in navigation, and navigation outcomes. The report also briefly described bridge organizations and their CDS partners' experiences with implementing screening, referral, and navigation. The AHC Model Evaluation report found that bridge organizations were engaged in staff training, however, training topics, competencies covered, and methodologies used to train staff varied by bridge organization and clinical delivery site (25). We further identified cross-cutting gaps in staff's core engagement competencies and boundary spanning skills, both of which are needed for an HRSN workforce, and which were not identified in the first model evaluation report. However, previous studies including a recent National Academies of Sciences, Engineering, and Medicine (NASEM) report have identified gaps in the knowledge base around the workforce engaged in HRSN intervention delivery, types of staff training, and skills needed for effective implementation and HRSN resolution (26). In a recent systematic review, only eight studies were identified that discussed skill-based staff training and none focused on boundary spanning (27). The identified gap in the workforce of boundary spanners for HRSN found in our study is important to further the field. Boundary spanners are organizational staff and leaders who are able to link their organization with its broader environment (28, 29), and in doing so, create not only connections and information transfer, but also build trust between actors in the intersectoral network (29, 30). This is particularly salient for staff trying to resolve HRSN, especially housing and transportation needs, since these needs cross community organization, clinical, and government silos. Future studies to identify key competencies of an HRSN workforce are needed.

In preparing a program champion, having engaged leadership facilitated a supportive environment for testing the model. Research has documented the critical role of leadership, from managers to the executive office, to provide visible support and engagement as facilitators for successful implementation (31–35). Leadership support is also often needed to facilitate cultural change and effective communication in organizational settings (31–35). In our study, BO staff perceived that turnover and hierarchical organizational structures were barriers to implementation as they presented challenges to maintaining effective communication. Leaders in the AHC Model BOs reported challenges in setting staff caseloads and goals for HRSN resolution. Reasons for this challenge are shown in our interview findings and align with the first AHC Model evaluation report, which showed the tension between delivering high-quality navigation services while balancing higher than expected caseloads (25). Currently, there are no standardized guidelines for leaders to set CHW (i.e., navigator) caseloads for HRSN. Literature available indicates a wide range of potential caseloads. Looking at recent HRSN studies and more established data from cancer navigation programs caseloads ranged from 55 to 300 per staff member a year (36, 37). The first year AHC Model evaluation report also indicated wide variation on the higher end of the spectrum, with caseloads averaging between 120 and 300 beneficiaries per navigator (25). Staff also reported a disconnect between those loads and model milestones for navigation, vs. their personal goal of resolving patient needs. This finding aligns with recent studies showing varying definitions of navigation “success” for HRSN (38). Similar to these studies, we found that staff documented a need resolved when a patient successfully received services and reported no longer needing navigator assistance. For leadership however, the AHC Model definitions required recording of multiple levels of resolution. A beneficiary's HRSN from navigation could be considered unreached if three attempts were made to contact the beneficiary, the HRSN case could be closed if community resources were deemed unavailable, and an HRSN was considered resolved-successful if a resource connection was made and it was perceived that the need could be met in 6 months, or resolved-resolved if the need was met. Future studies should consider standardizing definitions, caseloads, and program implementation guidelines for HRSN to provide leaders with standardized protocols which they can use to communicate and facilitate more effective implementation in the clinical setting. Similar to previous studies (24), in facilitation, we found that a history with similar programs made the AHC Model compatible and allowed managers to facilitate implementation in the clinical delivery setting based on their previous experience. The model further brought welcomed structure and accountability to implementation. Facilitation is an interactive process of problem-solving and support to address a recognized need (14). The disconnect between the recognized patient needs and staff goals was not adequately facilitated by management based on our findings.

We found that previous experience with data systems was a facilitator, while data integration across systems was a barrier. Recent studies have similarly identified the value of experience and having a champion for data collection (39). We identified barriers in available resources for data collection, including lack of integration between EHR and HRSN data systems and perceived duplication of effort resulting from this lack of integration. We identified barriers in data management related to using multiple systems. Previous studies have identified similar barriers in workflow optimization related to entering healthcare and HRSN data in multiple systems (39). Integration into the EHR, however, should not be seen as addressing all barriers at this time. Even within systems that have recorded HRSN data in the EHR, concerns over fragmentation of the data persist, with partial information housed in a variety of locations in the EHR, making action difficult (39). Further, barriers in workflow, data entry, and reporting have been reported similar to those found in our study despite using the EHR (39). Finally, the current lack of national standards for the collection, reporting, and sharing of this data present challenges and concerns both for beneficiaries (e.g., acceptability of screening and privacy) and health systems as vendors are currently determining implementation (39, 40).

In quality assurance, the AHC Model achieved mid-level integration with front-line staff and managers, adopting and testing a variety of improvement strategies, despite barriers of data system integration. However, the challenges with integration fed into barriers in quality assurance with a lack of readily available data to assess impact, return on investment, or the overall value of the AHC Model due to limited real-time data.

In community resource engagement (community alignment), a recurring barrier was alignment with CSPs, which crossed Characteristics of Individuals, Inner Setting, and Outer Setting domains. Barriers identified reflected the time BOs needed to engage site leadership, gaps in adequate training, and difficulty in establishing processes for advisory boards to aid in implementation, which were often interdependent. Studies of alignment have identified specialized expertise is key to an effective backbone (24, 41). We found no cross-cutting facilitators for alignment and wide variation in how BOs were implementing alignment activities. Engagement with advisory boards was difficult for BOs, who reported frequent turnover in CDS leadership attending the advisory board meetings, a finding similar to the first model report (25). For these BOs, the advisory board was mostly used to disseminate information. BO staff also identified concerns about the broader social safety-net and payment and other resources for CSPs to address HRSN in the 1-year time period. There are recently emerging concerns in the literature about anchoring HRSN interventions due to differences in power, priorities, capacity, funding, and the well-documented cost inefficiencies of the U.S. healthcare system (41, 42). We found that there were disconnects in priorities between the CDS and CSPs in our assessment. CDS were primarily concerned with patient model milestones and return on investment for conducting AHC activities, whereas CSPs were primarily concerned with resolving patient social needs even if beyond the 1-year navigation window. A recent study also found misalignment between CDS and CSPs on understanding of the demand for services, capacity to respond to HRSN and a lack of capacity building and alignment (43). While BOs in the AHC Model were aware of the disconnect, they were not able to effectively “bridge the gap,” partially due to lack of data, but also due to lack of organizational and staff capacity for alignment as an intervention.

### Future implementation process and research recommendations

Future implementers of HRSN interventions would benefit from applying implementation science approaches, including conducting a pre-implementation readiness assessment using a framework such as CFIR. Implementers would also benefit from conducting a planning review of potential ERIC strategies and matching implementation strategies to the readiness results. Choosing and measuring the implementation process and outcome measures which could be monitored over time is also important to support quality improvement.

Future research focused on identifying key competencies for a HRSN workforce is needed to help standardize and guide the field. Future studies using hybrid or adaptive trial designs that couple implementation with effectiveness would also be valuable. Future research should also explore the differing measurement priorities of clinical delivery sites and community service providers and test ways to align organizations across a range of measures.

### Limitations

Our study was limited to the five BOs who participated in the Strengthening Peer AHC Navigation (SPAN) model out of 28 BOs who participated in the AHC Model and should be interpreted accordingly. We also did not explicitly measure implementation outcomes in this study as these will be assessed as part of the model-wide assessment and are not yet available.

## Conclusion

We found the AHC Model was *compatible* with BOs and their previous experience and interest in HRSN. Variability of implementation was present, and barriers were identified in the implementation of all ERIC strategies.

## Data availability statement

The datasets presented in this article are not readily available because of restrictions in legal agreements related to data sharing. Requests to access the datasets should be directed to the corresponding author for consideration.

## Author contributions

LH conceived the study and led manuscript development. JH conducted the interviews. GF and JH contributed to and approved the manuscript. All authors participated in coding and interpretation. All authors contributed to the article and approved the submitted version.

## Funding

This study was supported by the Centers for Medicare and Medicaid Services (CMS) of the U.S. Department of Health and Human Services (HHS) as part of a financial assistance award totaling $529,632 with zero percentage funded by CMS/HHS and $424,000 amount and 100 percentage funded by nongovernment source(s), the Kresge Foundation and Episcopal Health Foundation. This work was supported by Kresge (grant number: 0015171) and EHF (grant number: 0015020).

## Conflict of interest

The authors declare that the research was conducted in the absence of any commercial or financial relationships that could be construed as a potential conflict of interest.

## Publisher's note

All claims expressed in this article are solely those of the authors and do not necessarily represent those of their affiliated organizations, or those of the publisher, the editors and the reviewers. Any product that may be evaluated in this article, or claim that may be made by its manufacturer, is not guaranteed or endorsed by the publisher.

## Author disclaimer

The contents are those of the author(s) and do not necessarily represent the official views of, nor an endorsement, by CMS/HHS, or the U.S. Government.
