# Supplementary material for: Barriers and facilitators to implementation of the Accountable Health Communities (AHC) Model: Findings from a between-site qualitative assessment of implementation strategies
Source: Front Health Serv. 2022 Nov 2;2:926657. doi: 10.3389/frhs.2022.926657 (PMC10012815; doi:10.3389/frhs.2022.926657)
Supplement: Supplementary file 1 [file Table_1.DOCX]

| **Expert Recommendation for Implementing Change (ERIC) strategies in AHC Model** | **Staff**  **training** | **Identify and prepare champions** | **Facilitation** | **Data**  **systems** | **Quality monitoring and assurance** | **Community resource engagement** |
| --- | --- | --- | --- | --- | --- | --- |
| **CFIR Domain** | Inner Setting | Intervention Characteristics | Intervention Characteristics | Intervention Characteristics | Inner Setting | Inner Setting |
| **CFIR construct** | Readiness for Implementation: Access to Knowledge & Information | Compatibility | Planning and Executing | Complexity | Climate: Goals & Feedback | Readiness for Implementation: Access to Knowledge & Information |
| **CFIR Domain** | Inner Setting | Inner Setting | Outer Setting | Inner Setting | Outer Setting | Outer Setting |
| **CFIR construct** | Available Resources | Networks & Communications | Patient Needs & Resources | Available Resources | Patient Needs & Resources | Patient Needs & Resources |
| **Theme - Barriers** | Gaining sustainable buy for the AHC Model from clinical delivery site staff | Justifying return of investment (ROI) of AHC | Adapting AHC screening questions or screening script while ensuring fidelity | Duplicating data entry across AHC and other data systems | Identifying successful patient contact and need resolution rates | Updating lists of community resources provided to patients |
| **Expert Recommendation for Implementing Change (ERIC) strategies in AHC Model** | **Staff**  **training** | **Identify and prepare champions** | **Facilitation** | **Data**  **systems** | **Quality monitoring and assurance** | **Community resource engagement** |
| **CFIR Domain** | Inner Setting | Inner Setting | Intervention Characteristics | Intervention Characteristics | Inner Setting | Inner Setting |
| **CFIR construct** | Readiness for Implementation: Access to Knowledge & Information | Climate: Learning Climate | Planning and Executing | Compatibility | Climate: Goals & Feedback | Leadership Engagement |
| **CFIR Domain** | Individual Characteristics | Inner Setting | Inner Setting | Intervention Characteristics | Inner Setting | Outer Setting |
| **CFIR construct** | Self-efficacy | Climate: Culture | Networks & Communications | Adaptability | Climate: Learning Climate | Patient Needs & Resources |
| **Theme - Facilitators** | Development of a robust training plan focused on the AHC Model and Model activities | Allocating time for innovation and wellness to support staff | Utilizing experts in specific client needs and populations for navigation activities | Applying skills and experience to utilize AHC data systems | Accessing monthly dashboards for monitoring and quality assurance | Engagement of community partners and patients in Advisory Board meetings |
